# Adherence to a Mediterranean Diet Pattern, Physical Activity, and Physical Self-Concept in Spanish Older Adults

**DOI:** 10.3390/nu14122404

**Published:** 2022-06-09

**Authors:** Javier Conde-Pipó, Cristina Bouzas, Félix Zurita-Ortega, Fátima Olea-Serrano, Josep A. Tur, Miguel Mariscal-Arcas

**Affiliations:** 1 Department of Didactics of Musical, Plastic and Corporal, Faculty of Education Sciences, University of Granada, 18071 Granada, Spain; javiercondepipo@gmail.com (J.C.-P.); felixzo@ugr.es (F.Z.-O.); 2 Research Group on Community Nutrition and Oxidative Stress, University of the Balearic Islands-IUNICS, 07122 Palma de Mallorca, Spain; cristina.bouzas@uib.es; 3 Health Institute of the Balearic Islands (IDISBA), 07120 Palma de Mallorca, Spain; 4 CIBEROBN (Physiopathology of Obesity and Nutrition CB12/03/30038), Instituto de Salud Carlos III (ISCIII), 28029 Madrid, Spain; 5 Department of Nutrition and Food Science, School of Pharmacy, University of Granada, 18071 Granada, Spain; folea@ugr.es (F.O.-S.); mariscal@ugr.es (M.M.-A.)

**Keywords:** Mediterranean diet, physical self-concept, physical activity, older adult

## Abstract

Background: The aging world population is accelerating rapidly. Physical self-concept (PSC) is one of the psychosocial factors with the greatest influence on an individual’s well-being and health. The traditional Mediterranean dietary pattern (MDP) is considered one of the healthiest dietary models, as it is nutritionally complete and easy to follow. Objective: To assess the adherence to MDP and its association with the practice of physical activity (PA) and PSC levels in the older adult Spanish population. Methods: A cross-sectional study was conducted on a representative sample of Spanish older adults (n = 342; older than 55 years old). Their PSC was assessed using a previously validated PSC questionnaire. Adherence to an MDP was assessed using a validated Mediterranean Diet Adherence Screener questionnaire. Their PA was measured using the Spanish version of the Rapid Assessment of Physical Activity Questionnaire. Data on age, sex, hypertension, cholesterol or diabetes suffered in the last 12 months, as well as weight, height, and BMI, were collected. Results: At the lowest levels of PSC, the percentage of individuals who were non-active and non-adhering to the MDP was lower compared to the highest levels (75.0% vs. 19.6; *p* = 0.001; Cramer’s V = 0.414, and 83.3% vs. 57.9%; *p* = 0.001; Cramer’s V = 0.221, respectively). This sample showed an abandonment of the most classic habits of the MDP, such as the consumption of olive oil, vegetables, fruits, nuts and fish. Conclusions: Non-adherence to the MDP and low levels of PA are associated with low levels of PSC in older adults.

## 1. Introduction

The aging world population is accelerating rapidly, driven by the decline in the birth rate and the increase in life expectancy, facilitated by socioeconomic progress and the most recent advances in medicine and public health [1,2]. Therefore, it is necessary to encourage the older adult population to adopt healthy lifestyles based on healthy nutrition and the practice of physical activity (PA) to minimize the risk of disease and disability. They can enjoy this greater longevity with a good quality of life while reducing the enormous costs of maintaining the public health and care services derived from this increased life expectancy [3,4,5].

Adequate nutritional status can play a key role in preventing or delaying the progression of age-related diseases such as cardiovascular diseases, cognitive function and osteoporosis [6,7,8,9,10]. However, this group is considered to be one of those at the highest risk of suffering from nutritional imbalances, deficiencies and problems. Age-related changes, such as decreased food intake, impaired sensory perception, malabsorption, difficulties in chewing and swallowing, declining activity and increased disability, have repercussions on the ability to feed oneself and the quality and balance of the diet [8,9,10,11]. The traditional Mediterranean dietary pattern (MDP) is considered one of the healthiest dietary models, as it is nutritionally complete, adequate and easy to follow. This is of the utmost importance for aging, mainly due to its benefits in increasing life expectancy, improving quality of life, and reducing the risk of suffering from cardiovascular and hepatic diseases, obesity, diabetes, osteoporosis, infections, tumours and inflammatory processes [6,10,12,13,14,15]. It also provides benefits for mental health, acting as a protective factor against mild cognitive impairment, dementia, Alzheimer’s, and depression, as well as being associated with better emotional functioning [5,7,16,17]. The MDP was the basis of food habits during the twentieth century in all countries of the Mediterranean region. However, adherence to the MDP has been progressively eroded due to recent changes in lifestyle [18]. This low MDP, together with a decrease in the practice of PA, is becoming a health problem, especially among the older population [18,19,20,21].

The benefits of PA are also numerous for both physical and mental health and are well described [22], being enough to practice moderately or vigorously for 150 min per week to obtain its beneficial effects [19,23,24]. Approximately 20–30% of premature mortality could be prevented through regular participation in PA [25].

Physical self-concept (PSC) is defined as the set of ideas and abilities that we believe define us physically [25,26]. It is comprised of four subdomains: physical condition, sports competition, physical attractiveness and strength [27,28]. Following the hierarchical model proposed by Shavelson [29], it is, in turn, part of the general self-concept, along with the academic, family, social and emotional dimensions. It is one of the psychosocial factors with the greatest influence on individual well-being and health since PSC is linked to healthy lifestyle habits and has proven to be inversely related to eating disorders [30].

There is a positive association between the practice of PA and PSC [3,27,31]. However, current research on adherence to the MDP and PSC has not focused on the older adult population. It has been pointed out there is a positive association between both, favoring Mediterranean diet parameters with an impact on PSC improvement [32]. Thus, low levels of PSC increase the risk of low adherence to the MDP in adolescents [30,32], while other evidence suggests that low adherence to the MDP can lead to increased incidence of overweight and, therefore, a lower level of PSC [33]. Nevertheless, the relationship between the MDP and body image, a variable analogous to the physical attractiveness dimension of the PSC, has been explored in depth, and the results can be extrapolated. Some research carried out with children and adolescents observed that a poor body image can lead to eating disorders [34,35,36]. Research developed with an older adult population indicated that a positive body image is associated with unhealthy dietary patterns, such as a higher intake of sweet beverages and refined foods or the abuse of supplementation. A reduced body image in this population is associated with a healthy dietary pattern [35,37], possibly because it corresponds to states of overweight or obesity and awareness. These people want to be and feel better [38]. Therefore, it is unclear what association exists between PSC and the MDP in older adults.

With the scientific literature reviewed and the insufficiency of the studies and the inconsistency of the results verified, this study aims to assess the adherence to the MDP and its association with the practice of PA and PSC levels in the older adult Spanish population.

## 2. Materials and Methods

### 2.1. Design and Subjects

The study design was cross-sectional, descriptive and comparative. According to the Spanish Statistic National Institute’s [39] (https://www.ine.es/, 4 June 2022) data on populations above 55 years old, it was estimated that a sample size of at least 273 participants would be sufficient under the conditions of α = 0.05 and a two-sided confidence interval = 90%. The sample was initially composed of 356 subjects, all Spanish from different regions, with a single inclusion criterion, namely, being at least 55 years old; 14 of them were discarded for not completing the questionnaires correctly, so the final sample was 342 subjects.

All subjects were recruited for three months, invited to participate voluntarily and provided written informed consent. They were informed of the objectives of this investigation, and data protection was ensured. The surveys were completed anonymously. The research complies with the principles of the Declaration of Helsinki and has the approval of the Research Ethics Committee of the University of Granada (Spain), with code 1230/CEIH/2020.

### 2.2. Outcomes

Sociodemographic data such as age, sex and more common diseases suffered in the last 12 months were collected (hypertension: systolic blood pressure ≥ 130 and diastolic ≥85 mmHg; cholesterol: HDL-c < 40 mg/dL (1.0 mmol/L) in men and <50 mg/dL (1.3 mmol/L) in women; diabetes: fasting glucose ≥ 100 mg/dL [40]). Anthropometric variables were taken by trained personnel. Height was measured in centimetres using a wall-mounted stadiometer (Seca 214, SECA Deutschland, Hamburg, Germany), and weight was measured in kilograms with a high-precision scale (Tanita BC-418, Tanita, Tokyo, Japan). All participants were weighed barefoot and wearing light clothing, subtracting 0.6 kg from the total for clothing. Body mass index (BMI) was calculated by dividing weight in kilograms by the square of height in meters (kg/m^2^).

Physical activity was measured using the Spanish version of the Rapid Assessment of Physical Activity Questionnaire (RAPA-Q) [41], a validated questionnaire that is easy to use, proven reliable and sensitive, and specifically designed for use with older adults [42]. Its seven items can be answered affirmatively (yes) or negatively (no) and allow easy identification of PA level (PAL), depending on whether the WHO minimum practice recommendations to obtain benefits for cardiovascular health [24] are achieved. Thus, the subjects who practiced more than 150 min per week of moderate activities or 75 min of vigorous activities were classified as active and the rest as non-active.

The Physical Self-Concept (PSC) was assessed using a PSC-Questionnaire (PSC-Q) that was an adaptation to the Spanish population [43] of the original Physical Self-Perception Profile (PSPP 30) [28]. It consists of 30 items that are valued on a five-point Liker scale (“Strongly disagree” = 1, “Strongly agree” = 5). It is divided into five dimensions: physical condition (PC, α = 0.84); sports competence (SC, α = 0.88); physical attractiveness (AT, α = 0.88); physical strength (ST, α = 0.83); and general physical self-concept (PSC, α = 0.88). These dimensions were categorized into quartiles of the score range of each test. Thus, the classes or levels-groups were “Very Low” (PC ≤ 10.50, AT ≤ 14.00, SC ≤ 10.50, ST ≤ 8.75, PSC ≤ 8.75); “Low” (PC ≤ 15.00, AT ≤ 20.00, SC ≤ 15.00, ST ≤ 12.50, PSC ≤ 12.50) “High” (PC ≤ 19.50, AT ≤ 26.00, SC ≤ 19.5, ST ≤ 16.75, PSC ≤ 16.75); and “Very High” (PC ≤ 24.00, AT ≤ 32.00, SC ≤ 24.00, ST ≤ 20.00, PSC ≤ 20.00).

To quantitatively estimate adherence to the MDP, the validated Mediterranean Diet Adherence Screener (MEDAS) [44] was used. This questionnaire was originally made from a validated FFQ (r = 0.52; *p* < 0.001) [44] and evaluated the effects of the MDP on the primary prevention of cardiovascular diseases [15,44,45,46]. It consists of 14 items, 12 on the frequency of consumption of main foods (olive oil, wine, fruits, vegetables, fish, legumes, nuts, meat and its derivatives, poultry, butter, pastries and carbonated/sweetened beverages), and 2 on the eating habits characteristic of the MDP: chicken and olive oil used as cooking fat. Each affirmative answer is valued with one point. If the sum of points is equal to or greater than 10, it was considered that there was adherence to the MDP [14], a fact that allowed the sample to be divided into two groups, those adhering to the MDP (AMD) and those who did not (NAMD).

### 2.3. Statistics

Statistical analysis was performed with the R statistical computing software (R Core Team, Vienna, Austria). The normality was analysed using the Kolmogorov–Smirnov test with the Lillieforts correction, and homoscedasticity was assessed with the Levene test. For the basic descriptions, frequencies, means and standard deviations were used. For comparisons between groups of continuous variables, the nonparametric Mann–Whitney U test for independent variables was used. For bivariate correlations, Spearman’s rho correlation coefficient was used. The association between categorical variables was evaluated using the Chi-square test and its magnitude with Cramer’s V coefficient. An effect size < 0.1 reflects no association, ≥0.1 but ≤0.3 reflects a weak association, >0.3 but ≤0.5 reflects a moderate association and >0.5 reflects a strong association. The internal reliability of the instruments was evaluated using Cronbach’s Alpha coefficient. All reported *p*-values are based on the two-tailed test, and the level of statistical significance for all tests was established at 95%. Any missing data involved the removal of study participants.

## 3. Results

Table 1 shows the characteristics of the sample according to their classification by sex, the PAL and adherence to the MDP. Significant differences were observed in all variables when comparing sexes, being higher in all values and proportions in men. No significant differences were obtained when comparing the AMD and the NAMD groups in any of the variables. Between the groups of active and non-active subjects, there were significant differences in the variable height (*p* = 0.032) and, consequently, BMI (*p* < 0.001). Regarding the practice of PA, the percentage of active subjects was higher than non-active (active = 68.24%, non-active = 31.75%; *p* < 0.001). Nevertheless, the percentage of subjects in the AMD group was lower than in the NAMD group (AMD = 39.88%, NAMD = 60.11%, *p* < 0.001).

Table 2 shows the bivariate correlations between the physical self-concept dimension, the adherence to the Mediterranean diet pattern, and physical activity. Significant and moderated correlations with the ADM was obtained by PSC (r = 0.27) and PC (r = 0.21). Regarding to PA, highlighted PC with a strong correlation (r = 0.46), followed by AT (r = 0.31) and SC (r = 0.31). No significant correlation was found between the MDP and PA.

Table 3 shows the distribution of the sample according to the results obtained in the PSC-Q classified in the level-groups (very low = VL; low = L; high = H; very high = VH), and differentiating between active/non-active and the ADM/NADM groups. The internal consistency of the PSC showed an acceptable level, both globally (α = 0.93) and for the different subdomains (SC, α = 0.86; PC, α = 0.87; AT, α = 0.81; ST, α = 0.72; PSC, α = 0.72). Significant differences were for all subdomains and groups except for the SC subdomain and MDP groups (*p* = 0.136). In all the PSC-Q subdomains, an increasing behaviour was observed in the proportion of subjects belonging to the AMD group from the categories VL to VH (PSC = 16.67% vs. 42.06%; PC = 22.73% vs. 43.38%; A = 29.03% vs. 45.79%; SC = 29.41% vs. 43.66%; ST = 30.43% vs. 47.66%), while for the NAMD group, this behaviour was inverse. The greatest differences regarding the percentage of subjects adhering to the MDP were obtained for the PSC dimension, where the VL category was composed of 83.33% of the NAMD and 57.94% of the AMD group (*p* = 0.001). This dimension was also the one that showed the strongest association with adherence to the MDP (Cramer´s V = 0.221), followed by PC (V = 0.173), ST (V = 0.155) and AT (V = 0.140). Regarding the practice of PA, significant differences (*p* < 0.001) were obtained between the categories of all the subdomains of the PSC-Q, also giving the highest percentages of the active population in the VH categories and the lowest in VL (PSC = 80.37% vs. 25.00%; PC = 89.71% vs. 29.55%; AT = 82.24% vs. 48.39%; SC = 77.46% vs. 41.18%; ST = 78.91% vs. 21.09%). The strongest associations occurred in the PC (V = 0.497) and the PSC (V = 0.414).

Table 4 shows the percentage of individuals who answered “yes” to each item of the MEDAS questionnaire, highlighting items 1, 5, 6, 7, 11, 13 and 14 for their high percentage (>70%), as well as the distribution for each level-group of PSC. In the highest level-group of PSC (VH), there was a higher proportion of individuals who stated that they consumed ≥4 tablespoons of extra virgin olive oil (EVOO) per day (*p* = 0.001, V = 0.206); ≥2 servings of vegetables per day, (*p* = 0.001, V = 0.300); ≥1 portion of red or processed meat per day (*p* = 0.029, V = 0.150); ≥3 portions of legumes per week (*p* = 0.001, V = 0.255); ≥3 fish servings per week (*p* = 0.001, V = 0.246); ≤2 sweets per week (*p* = 0.001, V = 0.212); and ≥3 servings of nuts per week (*p* = 0.001, V = 0.206). No significant association was found between PA practice and adherence to the MDP (active/ADM = 42.65% vs. non active/ADM = 33.92%; *p* = 0.898).

## 4. Discussion

The current results revealed that the PSC and its subdomains are positively associated with PA and MDP adherence in older adults. However, we hypothesized that this association with the MDP would be weaker than expected.

Regarding the positive association between PSC and the MDP, the current results are consistent with those reported by similar studies in the Spanish adolescent population [30,32,33,47,48]. It obtained very similar correlation values for PSC and the MDP [49]; however, it was also affirmed that self-concept in each dimension represents a significant risk of lack of adherence to the Mediterranean diet [30]. Moreover, the highest values of PSC were found in the high adherence group [33].

However, the association between the practice of PA and adherence to the MDP shown in recent studies [6,14,33,48] could not be corroborated. It could be incongruous since PSC is in line with what has already been established in the literature and positively and strongly associated with the practice of PA, especially in the subdomains PC and SC [30,46]. In the current study, all dimensions of PSC were positively associated with PA; these results are similar to previous research carried out with children and adolescents [30,31,47].

Nevertheless, in the current sample, there were considerable number of individuals with high or very high levels of PSC with high levels of the MDP but who were non-active. One of the explanations could lie in the age factor, since the older adult, despite being less active or practicing activity at very light intensities, tends to perceive himself positively and in good physical health when he maintains the strength, functionality and mobility necessary to perform daily tasks [50,51], or if that person was considered very active during the early stages of life, whether in childhood, adolescence or young adulthood [3].

Something similar occurs concerning physical attractiveness and body image since older adults tend to underestimate states of overweight or obesity, perceiving themselves as in good physical condition [11,37,38,48]. One of the strengths of the MDP is its strong cultural roots since it has been transmitted from generation to generation as part of the lifestyle of the Mediterranean area [18], being another reason that could reduce the differences between active and non-active individuals [37]. However, changes in most recent lifestyles are causing a progressive abandonment of the MDP that is already beginning to be evident even in precursor countries, such as Spain, Italy or Greece, in favour of a less varied diet and more Westernized [21,52]. In the coming years, the differences between active and non-active groups with regard to adherence to the MDP may be more pronounced, especially among generations after the well-known “baby boomers” [52].

It is striking that among all the items in the MEDAS questionnaire, there was a high prevalence for this population related to the low consumption of cream and butter, industrial pastries, carbonated beverages, and imported Western habits. These unhealthy habits are very popular today among the young population [52]. Still, some research found that adolescents who most practice physical activity also eat healthily, reporting a lower consumption of fast food and sweets [53].

Therefore, our results suggest that older adults know how to stay away from these habits. Nevertheless, they are losing others of vital importance for proper nutrition and health, such as the consumption of extra virgin olive oil (EVOO), vegetables, fruit, legumes, and nuts. This is especially notable in those individuals who showed a very low physical self-concept.

The current study shows that a situation of very low physical self-concept in older adults should be considered a risk factor for health since it was associated with less physical activity, with the consequences already described in the literature [22], and with a lower intake of foods typical of the Mediterranean diet that are of vital importance for the maintenance of a correct nutritional state. Specifically, the frequent consumption of EVOO, fish and nuts, foods rich in monounsaturated and polyunsaturated fatty acids, reduces both the risk of contracting a mental disability or dementia [7] and the risk of suffering cardiovascular events, some of which can seriously limit mobility and cause disability [45,54]. Fruits and vegetables, which are rich in magnesium and micronutrients with antioxidant and anti-inflammatory properties, benefit muscle metabolism, and their absence in the diet of the older adult is related to an increased risk of the loss of musculoskeletal health due to sarcopenia and osteoporosis [14,55,56], and with it, loss of quality of life due to the increased risk of fractures or physical disability [54]. Likewise, legumes, rich in vegetable proteins, fibre, antioxidants, phytochemicals and other bioactive components, improve glycaemic control, lower blood lipids, reduce intestinal fat absorption, and help reduce metabolic disorders [12].

### Strength and Limitations

The main strength of the current study is that it has been demonstrated that low levels of PA and non-adherence to the MDP are associated with low levels of PSC levels in older adults. The current study has several limitations. The main one is its descriptive cross-sectional design, which does not allow establishing causal relationships between the PSC, MDP and PA variables, so longitudinal studies will be required in the future. Secondly, the data obtained for the evaluation of both PA and dietary habits were reported by the participants. Lacking objective data, it is necessary that the data shown here and its interpretation be taken with caution.

## 5. Conclusions

Non-adherence to the MDP and low levels of PA are associated with low levels of PSC in older adults. The lack of adherence to the MDP is mainly reflected in a loss of classic habits of the MDP without including habits of globalization and those from other cultures. As a future perspective, it would be necessary to analyse the relationship between PSC both with the practice of PA and adherence to the MDP, including the possible influence of the state of health. Likewise, the design of educational and institutional health programs aimed at the older adult population should transmit and reinforce the importance for the health of keeping active and maintaining the eating habits typical of the Mediterranean diet so that they contribute to curbing its progressive abandonment.

## Figures and Tables

**Table 1:** Sample characteristics were classified by gender, physical activity level and adherence to the Mediterranean diet.

		All Sample (*n* = 342)			Physical Activity Level			Mediterranean Dietary Pattern		
		M	W	p *	Active	Non-active	*p* ^†^	AMD	NAMD	*p* ^†^
Distribution	*n*	258	84	-	222	120		144	198	
	%	75.44	24.56	0.001	64.91	35.09	0.001	42.11	57.89	0.001
Age (years)	Mean	61.31	62.36	0.119	61.84	61.05	0.081	61.56	61.57	0.417
	SD	5.69	5.87	-	5.67	5.87	-	5.36	6.02	-
	Median	60	60	-	60.00	59.00	-	60.00	60.00	-
	IQR	6.00	9.25	-	9.00	6.00	-	8.00	8.00	-
Height (m)	Mean	1.73	1.59	0.001	1.71	1.68	0.120	1.70	1.69	0.387
	SD	7.98	7.78	-	9.13	10.95	-	9.39	10.21	-
	Median	1.75	1.60	-	1.72	1.70	-	1.73	1.70	-
	IQR	10.00	9.00	-	13.00	18.00	-	12.25	15.00	-
Weight (kg)	Mean	80.68	66.07	0.001	75.89	79.04	0.153	76.24	77.60	0.677
	SD	11.36	12.25	-	11.22	15.93	-	11.86	14.06	-
	Median	79.00	63.00	-	75.00	78.00	-	75.00	77.00	-
	IQR	15.50	11.75	-	15.50	22.50	-	17.00	18.00	-
BMI (kg/m^2^)	Mean	26.88	25.89	0.014	25.95	27.83	0.009	26.13	26.98	0.845
	SD	4.34	4.94	-	3.54	5.67	-	3.87	4.90	-
	Median	25.95	24.60	-	25.24	26.43	-	20.69	21.82	-
	IQR	4.61	5.02	-	4.27	5.40	-	4.18	5.19	-
Hypertension	*n*	47	13	-	36	24	0.558	24	36	0.826
	%	18.21	15.47	0.682	16.21	20.00	-	18.18	16.66	-
Diabetes	*n*	14	2	-	9	7	0.635	8	8	0.692
	%	5.42	0.23	0.391	4.05	5.83	-	4.04	5.55	-
Cholesterol	*n*	58	28	-	40	22	1.000	20	42	0.111
	%	15.50	26.10	0.040	18.01	18.33	-	13.88	21.12	-

Abbreviations: M (men), W (women); AMD (adherence to the Mediterranean diet); NAMD (non-adherence to the Mediterranean diet); IQR (interquartile range); SD (standard deviation). * By U Mann–Whitney test; ^†^ by Chi-square test.

**Table 2:** Bivariate correlations between the physical self-concept´s dimension, adherence to Mediterranean diet pattern, and physical activity.

Variable	PSC	PC	AT	SC	ST	MDP
PC	0.50 **					
	(0.42, 0.50)					
AT	0.60 **	0.64 **				
	(0.52, 0.66)	(0.58, 0.70)				
SC	0.56 **	0.72 **	0.52 **			
	(0.48, 0.62)	(0.66, 0.77)	(0.44, 0.60)			
ST	0.53 **	0.66 **	0.60 **	0.59 **		
	(0.45, 0.61)	(0.59, 0.72)	(0.52, 0.66)	(0.52, 0.66)		
MDP	0.27 **	0.21 **	0.14 *	0.13	0.19 **	
	(0.07, 0.37)	(0.10, 0.30)	(0.14, 0.24)	(0.03, 0.20)	(0.09, 0.29)	
PA	0.23 **	0.46 **	0.31 **	0.31 **	0.26 **	0.08
	(0.12, 0.33)	(0.37, 0.54)	(0.21, 0.40)	(0.21, 0.41)	(0.16, 0.35)	(−0.03, 0.18)

Abbreviations: PSC (general physical self-concept); PS (physical condition); SC (sport competence); AT (physical attractiveness); ST (strength); PA (physical activity); MDP (Mediterranean diet pattern); * *p* < 0.05; ** *p* < 0.01; by Spearman’s rho correlation test.

**Table 3:** Association between level-groups of each PSC-Q dimension with physical activity and adherence to the Mediterranean diet pattern.

Variable and Groups	Very Low	Low	High	Very High		
	%	%	%	%	*p* *	Cramer´s V
	General Physical Self-Concept					
Physical Activity Level						
Active	25.00	66.67	59.74	80.37	0.001	0.414
Non-active	75.00	33.33	40.26	19.63		
Mediterranean Diet						
AMD	16.67	41.67	38.53	42.06	0.001	0.221
NAMD	83.33	58.33	61.47	57.94		
	Physical Condition					
Physical Activity Level						
Active	29.55	49.14	80.46	89.71	0.001	0.497
Non-active	70.45	50.86	19.54	10.29		
Mediterranean Diet						
AMD	22.73	38.86	42.53	43.38	0.007	0.173
NAMD	77.27	61.14	57.47	56.62		
	Physical Attractiveness					
Physical Activity Level						
Active	48.39	52.38	74.18	82.24	0.001	0.298
Non-active	51.61	47.62	25.82	17.76		
Mediterranean Diet						
AMD	29.03	33.33	42.62	45.79	0.049	0.140
NAMD	70.97	66.67	57.38	54.21		
	Sport Competence					
Physical Activity Level						
Active	41.18	59.09	83.85	77.46	0.001	0.351
Non-active	58.82	40.91	16.15	22.54		
Mediterranean Diet						
AMD	29.41	38.89	43.23	43.66	0.136	0.118
NAMD	70.59	61.11	56.77	56.34		
	Strength					
Physical Activity Level						
Active	43.48	57.36	70.68	78.91	0.001	0.278
Non-active	56.52	42.64	29.32	21.09		
Mediterranean Diet						
AMD	30.43	30.23	41.77	47.66	0.022	0.155
NAMD	69.57	69.77	58.23	52.34		

Abbreviations: AMD (adherence to Mediterranean diet); NAMD (non-adherence to Mediterranean diet); PSC-Q (Physical Self-Concept Questionnaire). * By Chi-square test.

**Table 4:** Association between items of MEDAS and level-groups of physical self-concept.

	MEDAS Questionnaire	Physical Self Concept by Level-Groups						
		All Sample	Very Low	Low	High	Very High		
Item	Question	% Yes	% Yes	% Yes	% Yes	% Yes	*p* *	Cramer´s V
1	Use of EVOO as main culinary lipid	98.48	100	98.61	99.57	97.20	0.256	0.101
2	EVOO > 4 tablespoons	50.47	33.33	52.78	51.52	61.68	0.001	0.206
3	Vegetables ≥ 2 servings/day	40.07	8.33	37.50	38.53	44.39	0.001	0.300
4	Fruits ≥ 3 servings/day	45.74	41.67	45.83	41.99	50.00	0.604	0.068
5	Red/processed meats < 1/day	80.71	75.00	68.06	81.82	84.11	0.029	0.150
6	Butter, cream, margarine < 1/day	95.08	100	94.44	94.37	95.79	0.128	0.119
7	Soda drinks < 1/day	89.79	91.67	90.28	90.04	89.25	0.950	0.030
8	Wine glasses ≥ 7/week	18.90	8.33	16.67	22.08	16.82	0.065	0.134
9	Legumes ≥ 3/week	48.58	25.00	59.72	45.02	50.00	0.001	0.255
10	Fish/seafood ≥ 3/week	42.91	16.67	44.44	41.13	45.79	0.001	0.246
11	Commercial sweets < 2/week	78.07	58.33	80.56	81.82	74.30	0.001	0.212
12	Tree nuts ≥ 3/week	55.38	33.33	52.78	51.52	61.68	0.001	0.206
13	Poultry more than red meats	75.42	83.33	80.56	76.19	72.43	0.256	0.101
14	Use of sofrito sauce ≥ 2/week	71.83	66.67	65.28	71.43	74.77	0.439	0.082

Abbreviations: EVOO (extra virgin olive oil); MEDAS (Mediterranean Diet Adherence Screener). * By Chi-square test.

## Data Availability

There are restrictions on the availability of data for this trial due to the signed consent agreements around data sharing, which only allow access to external researchers for studies following the project’s purposes. Requestors wishing to access the trial data used in this study can make a request to pep.tur@uib.es.
